# Female gorillas form highly stable dominance relationships

**DOI:** 10.1098/rsbl.2024.0556

**Published:** 2025-01-22

**Authors:** Nikolaos Smit, Martha M. Robbins

**Affiliations:** ^1^ Max Planck Institute for Evolutionary Anthropology, Deutscher Platz 6, Leipzig 04103, Germany

**Keywords:** female–female dominance relationships, stability, hierarchy, rank, gorillas

## Abstract

Animals commonly form dominance relationships that determine the priority of access to resources and influence fitness. Dominance relationships based on age, immigration order or nepotism (alliances with kin) conventions are usually more stable than those based on intrinsic characteristics such as physical strength. Unlike most mammals, female gorillas disperse from their groups, typically more than once in their lifetimes, disrupting their group tenures and/or any alliances. Thus, we predicted that they form unstable dominance relationships that are not based on conventions. Contrarily, using a 24-year dataset on five groups of both gorilla species, we found that females form strikingly stable dominance relationships, maintained over their whole co-residence in a group (mean dyadic co-residence = 4.8, max = 17.3 years). Specifically, we observed rank reversals in only two out of 92 female dyads, and all other rank improvements resulted from emigration or death of higher-ranking females (passive mobility). These results mirror observations in chimpanzees, suggesting that dominance dynamics might have deep roots in hominid evolution. Our study challenges a hypothesized link between hierarchy instability and dispersal, particularly among animals in which fitness consequences of rank improvement may not be great enough to counterbalance the potentially high costs of challenging higher-ranking individuals.

## 1. Introduction

Animals typically compete with conspecifics and can form dominance relationships that determine the priority of access to resources while minimizing repeated contests [1–4]. Dominance relationships can be based on fighting ability [4], or on age [5], immigration order [6], or nepotism (alliances with kin) [7] conventions. When individuals improve their ranks through fights against higher-ranking individuals (active mobility), dominance hierarchies may be relatively unstable [4]. In contrast, in convention-based hierarchies, such as the age-based female hierarchies in some ungulates [5,8] or the nepotism-based female hierarchies in many primates [7,9], relationships are commonly highly stable and rank improvements depend on death or emigration of higher-ranking individuals (passive mobility).

The stability of dominance relationships can be influenced by various other factors. First, the formation of coalitions with non-kin, although rarer than alliances with kin, can reinforce hierarchical relationships [10,11]. Second, the operation of ‘winner/loser effects’ can promote relationship stability when previous outcomes of conflicts result in ‘winners’ winning and ‘losers’ losing again [12,13]. Third, resource scarcity and monopolizability of resources may impact contest competition, which may, in turn, influence the stability of dominance relationships in both directions: it may reinforce agonism from higher- to lower-ranking individuals that increases relationship stability [14] or, contrarily, it might prompt low-ranking individuals to challenge higher-ranking ones to obtain crucial resources [15,16], destabilizing dominance relationships.

Dispersal patterns may also influence the stability of dominance relationships through their influence on group tenure and alliances. In species with sex-biased dispersal, different factors may influence the dominance relationships within each sex. Dispersal may disrupt any alliances or winner/loser effects and immigrants are expected to base their rank acquisition potential on their physical strength [17]. Thus, members of the dispersing sex are expected to be less socially integrated, profit little from social support and form less stable dominant relationships than members of the philopatric sex that often form convention-based dominance relationships [18–21]. In mammals, where female philopatry and male dispersal are the most common patterns [22], female hierarchies are expected to be convention-based while male hierarchies are expected to be based on fighting ability [4].

In contrast to most mammals, female gorillas typically disperse from their natal groups and often disperse again between groups (secondary dispersal), so their ranks are not maternally inherited [23,24]. Accordingly, they form relatively weak affiliative associations with other females [25] and in the rare events of female–female coalitions against other females, males may intervene to disrupt these coalitions [26,27]. Based on these traits, we would predict female gorillas to form unstable dominance relationships with common rank reversals [4,14]. However, previous studies have yielded mixed results regarding female gorilla hierarchy stability [28–32]. Notably, these studies were typically conducted over short time periods, often included data from only one group and used various static (matrix-based) methods that provided only final individual ranks that ignore interaction sequence, making it difficult to distinguish the influence of demographic changes and active challenges on rank reversals/improvements (but see [33]). Here we use a consistent methodology and longitudinal behavioural interactions between 2000 and 2023 from one western (*Gorilla gorilla gorilla*) and four mountain (*Gorilla beringei beringei*) gorilla groups to compare the hierarchy stability in the two species and test if female gorillas form unstable hierarchical relationships or, instead, stable relationships based on immigration order or another convention.

## 2. Methods

### (a) Study system and behavioural data

**Figure 1.**
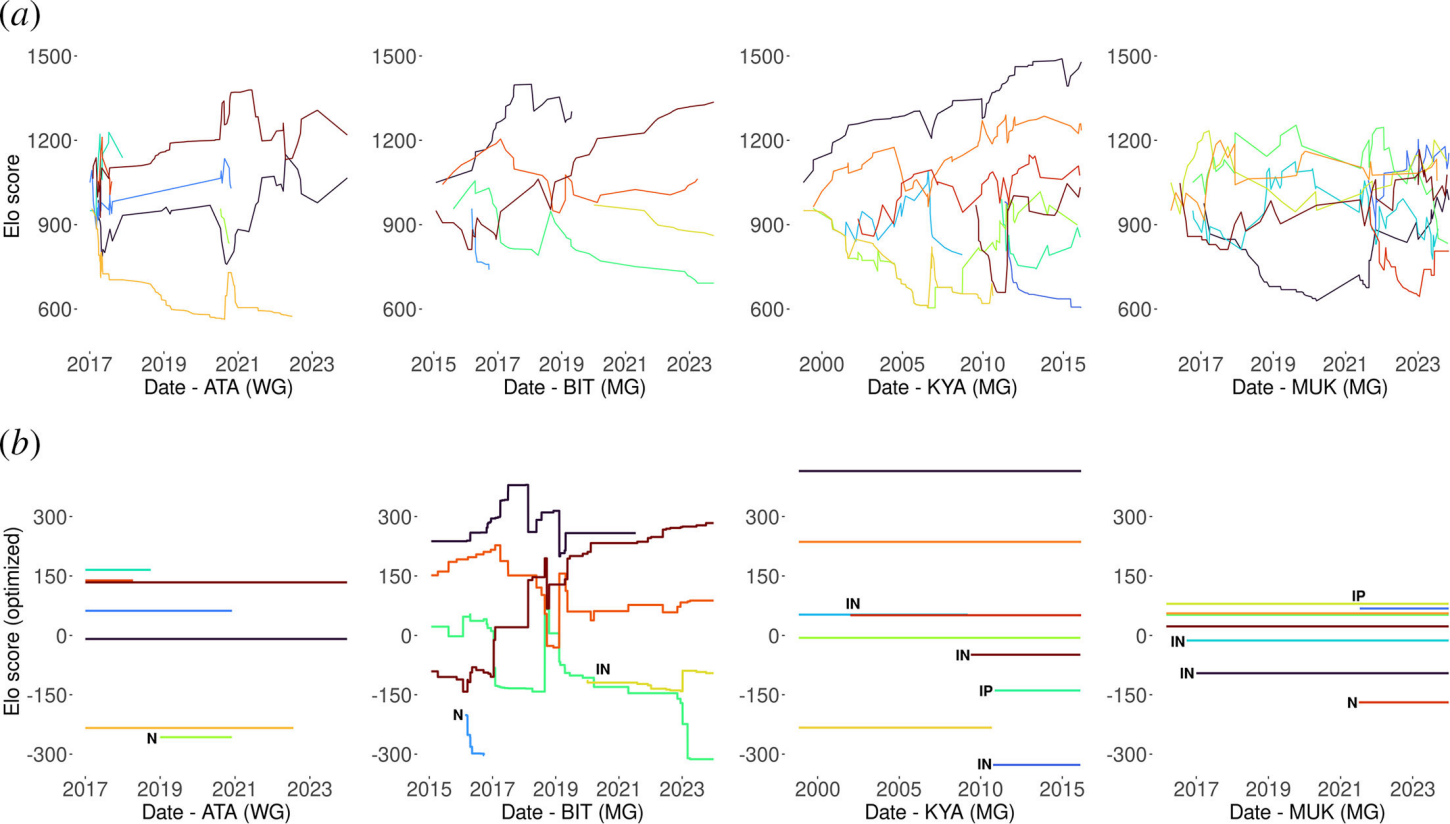
Female rank trajectories throughout the study period: Elo scores based on (*a*) the traditional Elo rating method and (*b*) the optimized Elo rating method. Each panel represents one group and each coloured line represents an individual. Within each group, an individual has the same colour in the two panels. Due to the fit of initial scores, the optimized method infers negative scores for some low-ranking individuals. In contrast, the traditional method, where initial scores are set to a large positive value (1000), infers only positive scores. In (*b*), females who entered the hierarchy when they reached maturity in their natal groups are marked with an ‘N’ (natal; three females), those who immigrated in a group while nulliparous and entered the hierarchy soon after are marked with an ‘IN’ (immigrant nulliparous; six females) and those who immigrated in a group while parous and immediately entered the hierarchy are marked with an ‘IP’ (immigrant parous; two females). ‘WG’ stands for ‘western gorillas’ and ‘MG’ for ‘mountain gorillas’

We studied one western lowland gorilla group in Loango National Park, Gabon (group ATA/Atananga, 7 years of data, seven females) where observations lasted typically between 07.00 and 16.30. We also studied four mountain gorilla groups in Bwindi Impenetrable National Park, Uganda (BIT/Bitukura, 10 years, six females; KYA/Kyagurilo, 17 years, nine females; MUK/Mukiza, 8 years, eight females; ORU/Oruzogo, 8 years, nine females; figure 1) where observations were limited to 4 h per day, typically between 08.00 and 15.00 following the regulations of the Uganda Wildlife Authority.

Observers blind to the topic of this study collected data on the five gorilla groups. They used both ad libitum and focal animal observations (15–60 min; [34]) to collect behavioural data. They specifically recorded avoidance (when an individual walks away from another approaching individual) and displacement (when an individual avoids another and the latter takes the place of the first) interactions. We used these interactions to infer social hierarchies (as per [29,33]) and we did not use aggression, as the expression of aggression might depend on context or incentives and may not accurately reflect hierarchical relationships as ‘ritualized’ and decided interactions do [35,36].

We included only adult females in the hierarchies (i.e. at least 8-years-old mountain gorilla females and at least 10-years-old western gorilla females [37]). The estimates of the age of females who were already parous at the beginning of the study were not very accurate, so we did not use female age as a covariate to explain dominance relationships. However, we draw a distinction between females who reached maturity in their natal group and did not disperse (figure 1*b*; ‘N’), females who immigrated into a group shortly before or after ‘adulthood’, while they were nulliparous and they likely had not yet reached their full adult body size (between 6- and 9-years-old; most likely natal dispersal; figure 1*b*; ‘IN’; [38]) and females who immigrated into a group as adults and parous (two females, more than 14-years-old; most likely secondary dispersal; figure 1*b*; ‘IP’).

### (b) Analysis

We performed all analyses in R version 4.1.2. We inferred daily individual Elo scores, corresponding to individuals’ ranks, using a widespread (traditional) Elo rating method ([39,40]; function elo.seq, R package EloRating; [41]). We assigned to all individuals present at the beginning of the study period an initial Elo score of 1000 and to individuals entering the hierarchy later (e.g. female reaching maturity or immigrants) the score of the lowest-ranking individual during the entrance day, because maturing individuals and immigrants are often thought to be subordinate to other group members [29,42]. After each interaction, the Elo score of the winner and loser increases and decreases, respectively, as a function of constant *k* and the winning probabilities prior to the interaction: the smaller the winning probability of the loser, the smaller the scores’ changes. We assigned equal intensity (*k* = 100) to all interactions. The calculation of Elo scores after each interaction provides daily ranks, allowing to distinguish between active and passive hierarchical mobility. Active mobility is characterized by rank changes due to agonistic interactions, and passive mobility is characterized by rank changes due to the removal (death or emigration) of individuals from the hierarchy.

To ensure the robustness of our results, we also used an optimized version of the above method, which is not yet widely used. This method (function eloratingopt, R package EloOptimized; [43,44]) implements maximum likelihood fitting of the constant *k* and the individuals’ initial Elo scores when entering the hierarchy. In contrast to the traditional Elo rating method, it can control for the effect of arbitrary initial rank assignments and the over- or underestimation of the *k* constant that determines rank changes after each interaction (for details see [43]). This method includes in the hierarchy only individuals who have at least one win and one loss during the study period. As a result, two females (one in ATA and one in BIT) that never won or lost any interaction were not included in the hierarchies. For another four females (one in ATA, two in BIT and one in KYA) that interacted agonistically but had only losses (minimum number of interactions = 7), we added dummy winning interactions at the end of the study period, so that the algorithm includes them in the hierarchy. In these dummy interactions, we specified as losers the lowest-ranking females in the hierarchies inferred without dummy interactions. Importantly, the inferred hierarchies were qualitatively similar with or without the inclusion of these females/dummy interactions, that is, the dominance relationships of all other females were not influenced by the dummy interactions.

We used the function stab_elo from the R package EloRating to calculate the S index, which represents the overall stability of a hierarchy (based on the traditional Elo rating method), that is, the frequency of rank reversals. The S index ranges from 0, reflecting absolute instability (complete reversal of ranks every other day), to 1, reflecting absolute stability and lack of rank reversals.

The number of displacement/avoidance interactions varied largely in the five study groups (ATA: 143 interactions, 20.4 ± 18.5 (mean ± s.d.) per year; BIT: 77, 8.6 ± 5.3; KYA: 199, 10.5 ± 11.0; MUK: 152, 19.0 ± 10.9; ORU: 32, 3.6 ± 2.1). Notably, we did not infer a hierarchy for the group ORU due to the very low sample size. The small number of recorded interactions is unlikely to be exclusively due to low sampling effort, as group ORU contained on average, 4.5 ± 1.7 (± s.d.) females and it was observed for 10 years (2014−2024; 6737 h of observation). Instead, the lack of interactions might reflect weak dominance relationships [28].

## 3. Results

The standardized Elo scores inferred from the optimized method were very strongly correlated with those inferred from the traditional method (Spearman rank test: *ρ* = 0.87, *p* < 0.001; see also figure 1), suggesting that the dominance relationships inferred from the two methods are very similar and validating the use of the optimized method. In the following paragraphs, we focus on the results of the optimized method. Given that the traditional Elo rating method uses a constant *k* = 100, all interactions led to changes in Elo scores and produced more ‘noisy’ rank trajectories (figure 1*a*) than those inferred from the optimized method (figure 1*b*).

The inferred dominance hierarchies of female gorillas were strikingly stable, with an average stability index equal to 0.99 (1 reflects absolute stability; $S_{ATA}=0.998$, $S_{KYA}=0.999$, $S_{BIT}=0.999$ and $S_{MUK}=0.997$; traditional method), highlighting the rarity of rank reversals. Accordingly, the optimized method showed that, of the 92 female dyads, active rank reversals occurred in only two (figure 1*b*; group BIT) and thus most females maintained stable rank relationships throughout an average group co-residence of 4.8 years (ATA: mean ± s.d. = 2.68 ± 1.75, max = 6.95 years; BIT: 4.34 ± 3.23, 8.7; KYA: 8.83 ± 4.28, 17.28; MUK: 4.97 ± 2.52, 7.69). Specifically, 16 relative rank improvements (10 in ATA, four in BIT and two in KYA) occurred due to death or emigration of higher-ranking individuals.

The estimated k constant (optimized Elo-rating method), representing the change of interacting individuals’ ranks after each interaction, was virtually equal to 0 in three out of four groups. Therefore, the rank scores did not change as a result of agonistic interactions. In the fourth group where the two rank reversals were observed (BIT; mountain gorillas), the k constant was estimated to be 122.25; suggesting that the inferred hierarchy stability in the other three groups, is not just an artefact due to small sample sizes (the smallest number of interactions was observed in BIT).

In total, 11 females entered a group’s hierarchy after the beginning of the study: three when they reached maturity in their natal group (figure 1*b*; ‘N’), six when they immigrated into the group between ages 6.4 and 8.8 years, while nulliparous and before reaching their full adult body size (figure 1*b*; ‘IN’), and two when they immigrated into the group as adults and parous (more than 14 years old; figure 1*b*; ‘IP’). Seven of these females entered at the bottom of the hierarchy. The remaining four females obtained a rank higher than (several) females already present in the hierarchy (figure 1*b*; three females that immigrated while nulliparous: yellow in BIT, red in KYA and brown in KYA; one parous immigrant: blue in MUK). Therefore, female gorillas did not follow a strict rank attainment convention based on the order of immigration (figure 1*b*). This result suggests that the traditional Elo rating method may be based on an oversimplification when assigning the score of the lowest-ranking individual on the entrance day to entering individuals.

Group MUK was formed after a fission of group KYA in 2016. Interestingly, the three highest-ranking females of the group remained with the original alpha male (that group was not included in this study because two of those females emigrated to an unhabituated group 11 months after the fission) and the four lowest-ranking females remained with another male to form MUK. This observation suggests that female rank may influence dispersal or fission decisions (the four females in MUK had greater relative ranks in comparison to their time in KYA). Interestingly, the dominance relationships among the four females were different in KYA (rank order: MG > TW > KR > JN) and MUK (rank order: KR > MG > JN > TW), suggesting that female–alpha male relationships potentially influence female–female dominance relationships [45].

## 4. Discussion

Our study challenges a hypothesized link between hierarchy instability and dispersal. Despite female dispersal typically occurring more than once in a lifetime, females showed exceptionally stable dominance relationships in both gorilla species. Furthermore, despite the differences in social organization and feeding ecologies of the two species [46], we found dominance relationships of female western gorillas to be similar to those of female mountain gorillas. The question of what maintains this remarkable female hierarchy stability emerges. First, gorillas reside in relatively small groups (average number of females in our study groups: 5.2; range: 2−8), a trait linked to stable hierarchies in other species [4,47]. Second, high-ranking individuals might use aggression towards lower-ranking ones to reinforce hierarchical relationships similar to other species [37,48,49]. However, female gorillas direct only around 60% of aggressive interactions down the hierarchy [15,28], suggesting that this hypothesis might have a smaller predictive power in these species.

The observed hierarchy stability suggests that active mobility appears to play only a small role in the formation and maintenance of female gorilla dominance relationships. Instead, rank improvements were usually the result of passive mobility occurring due to the death or emigration of higher-ranking females; additionally, a group fission improved the relative rank for three females. Similar stable dominance relationships and passive hierarchy mobility are observed in female chimpanzees (*Pan troglodytes schweinfurthii*) [43], suggesting that these traits might have been shared among early hominids. The formation and maintenance of stable dominance hierarchies can save energy, reduce stress and ultimately increase fitness [50,51]. Thus, the fitness benefits of rank improvement [52,53] may not be great enough to counterbalance the potentially high costs of challenging higher-ranking individuals [54]. This may promote the development of stable dominance hierarchies, especially among females, whose reproductive success is often more strongly influenced by reproductive lifespan rather than high rank [55–57]. In contrast, male reproductive skew is more strongly linked to dominance rank, and thus, active rank acquisition can increase male lifetime reproductive success significantly [58,59] despite the associated costs (e.g. injuries).

Female gorillas did not always enter at the bottom of their hierarchies upon immigration, meaning that the observed hierarchy stability does not result from a strict immigration order convention [29]. Their ‘entry ranks’ are likely influenced by other factors that can accelerate the acquisition of greater status and finally increase reproductive success. Our understanding of the potential determinants of these entry ranks, such as social support (spotted hyenas, *Crocuta crocuta*: [6]), age (hover wasps, *Liostenogaster flavolineata*; [60]) or body size (clownfish, *Amphiprion percula*; [61]) across species is limited.

Observations of captive female western gorillas suggest that although rare, social support, particularly among kin, may indeed influence dominance rank [62], similar to female chimpanzees [43]. Female resident chimpanzees or gorillas might improve the entry ranks of their maturing daughters and impair those of unrelated immigrants by being particularly aggressive to these immigrants [37,63]. Yet, all three natal female gorillas entered at the bottom of the hierarchy upon reaching maturity, not supporting this hypothesis. Six nulliparous females who immigrated into the study group were not yet fully adult size (M.M.R., personal observation; [38]), which may influence their competitive ability with resident adult females and explain why most of them also enter at the bottom of the hierarchy. However, previous work in wild mountain gorillas in the Virunga Volcanoes [29,33] and captive western gorillas [42] showed that female rank is correlated with age but not body size. These latter results are at odds with theoretical expectations: dominance relationships among the members of the dispersing sex are more likely to be influenced by body size rather than age (§1). A speculative explanation could suggest that (alpha) males intervene in female–female contests in favour of older or smaller females. Older females might have a greater probability of successful reproduction than young primiparous females [64] and are more likely to have already reproduced with the male, who might increase his fitness (e.g. offspring survival) by favouring these females. Males might also support smaller, physically weaker females to prevent bigger females from expelling the former from the group, mitigating the effect of female body size on female–female dominance relationships.

Overall, our study shows that the link between hierarchy instability and dispersal is not universal—or it might even be uncommon in species such as great apes where fitness gains of improved rank may be outweighed by potential costs of challenging the hierarchy. Females of these species appear to maintain the ranks they obtain upon entrance in a group’s hierarchy, resulting in strikingly stable dominance relationships over the whole duration of female–female co-residency. Future work may examine the factors that determine how rank is obtained upon immigration or maturation and quantify any fitness benefits of this stability.

## Data Availability

The data and code necessary to replicate this study are available from Zenodo [65].
